# Satellite DNA in Neotropical Deer Species

**DOI:** 10.3390/genes12010123

**Published:** 2021-01-19

**Authors:** Miluse Vozdova, Svatava Kubickova, Natália Martínková, David Javier Galindo, Agda Maria Bernegossi, Halina Cernohorska, Dita Kadlcikova, Petra Musilová, Jose Mauricio Duarte, Jiri Rubes

**Affiliations:** 1 Department of Genetics and Reproductive Biotechnologies, Central European Institute of Technology—Veterinary Research Institute, Hudcova 70, 621 00 Brno, Czech Republic; kubickova@vri.cz (S.K.); cernohorska@vri.cz (H.C.); kadlcikova@vri.cz (D.K.); musilova@vri.cz (P.M.); rubes@vri.cz (J.R.); 2 Institute of Vertebrate Biology, Czech Academy of Sciences, Kvetna 8, 603 65 Brno, Czech Republic; martinkova@ivb.cz; 3 Deer Research and Conservation Center (NUPECCE), School of Agricultural and Veterinarian Sciences, São Paulo State University (Unesp), 14884-900 Jaboticabal, Brazil; dgalindoh89@gmail.com (D.J.G.); agm.bernegossi@gmail.com (A.M.B.); mauricio.barbanti@unesp.br (J.M.D.)

**Keywords:** Cervidae, comparative cytogenetics, FISH, satellite DNA, sequencing

## Abstract

The taxonomy and phylogenetics of Neotropical deer have been mostly based on morphological criteria and needs a critical revision on the basis of new molecular and cytogenetic markers. In this study, we used the variation in the sequence, copy number, and chromosome localization of satellite I-IV DNA to evaluate evolutionary relationships among eight Neotropical deer species. Using FISH with satI-IV probes derived from *Mazama gouazoubira*, we proved the presence of satellite DNA blocks in peri/centromeric regions of all analyzed deer. Satellite DNA was also detected in the interstitial chromosome regions of species of the genus *Mazama* with highly reduced chromosome numbers. In contrast to *Blastocerus dichotomus*, *Ozotoceros bezoarticus*, and *Odocoileus virginianus*, *Mazama* species showed high abundance of satIV DNA by FISH. The phylogenetic analysis of the satellite DNA showed close relationships between *O. bezoarticus* and *B. dichotomus.* Furthermore, the Neotropical and Nearctic populations of *O. virginianus* formed a single clade. However, the satellite DNA phylogeny did not allow resolving the relationships within the genus *Mazama*. The high abundance of the satellite DNA in centromeres probably contributes to the formation of chromosomal rearrangements, thus leading to a fast and ongoing speciation in this genus, which has not yet been reflected in the satellite DNA sequence diversification.

## 1. Introduction

Among large mammals, Neotropical deer (Cervidae, Pecora, Ruminantia, Artiodactyla) [1,2] represent an interesting group of species still lacking comprehensive scientific data. Their taxonomy has been established mostly on the basis of morphology indicating a need for its critical revision and a future systematic research [3,4]. Neotropical deer involve genera *Pudu*, *Mazama*, *Hippocamelus*, *Blastocerus*, *Ozotoceros*, and *Odocoileus* grouped in the tribe Rangiferini, subfamily Capreolinae [4,5]. As in other Cervidae, a variety of karyotypes has been observed in Neotropical deer, ranging from 2n = 70 in *Mazama gouazoubira* or *Odocoileus virginianus*, to 2n = 32–34+ Bs in *Mazama bororo* [6,7]. The 2n = 70 karyotype considered to reflect cervid ancestral karyotype has derived from the hypothetical ancestral karyotype of Pecora (2n = 58) by six chromosome fissions [6,8,9]. However, a series of evolutionary chromosome rearrangements occurred in many deer taxa, which led to a significant diversification of their karyotypes [9]. As with the other Neotropical deer species, a rapid karyotype evolution has been observed in *Mazama americana*, a taxon grouping several cryptic species currently classified as cytotypes on the basis of their karyotype differences and geographical distribution and reported as *M. americana* species complex [4,10,11,12]. There is no doubt that the taxonomy and phylogenetics of the Neotropical deer would benefit from new approaches and utilization of new molecular markers.

A useful source of information can be found in satellite DNA, which consists of rapidly evolving, tandemly organized repetitive sequences, and might serve, to some extent, as molecular cytogenetic marker to trace individual and species origin and phylogeny. Satellite DNA located in centromeres and pericentromeric chromosome regions probably represents a structure linked to centromeric functions and chromosome segregation [13,14,15,16]. However, the functional roles of satellite DNA have not been fully elucidated yet. It is known that despite the relative uniformity of monomer lengths within satellite DNA families, they often show variations in sequence, copy numbers, and chromosome distribution even among related species [13,17], which can be used in phylogenetic studies [16,18,19,20,21,22,23,24,25,26,27].

Six satellite DNA families were described in Cervidae so far, of which satI-satIV were characterized in terms of sequence and chromosomal distribution in a number of Eurasian and North American cervid species [24,28,29,30,31,32,33,34]. However, there is a complete lack of data on satellite DNA sequences and their chromosome distribution in deer inhabiting South America. The sole exception is *O. virginianus*, a species spread throughout the American continent, in which a representative of its northern population has been under study recently [24].

In this study, we isolated four main groups of cervid satellite DNA sequences (satI-IV) in eight Neotropical deer species: *Mazama gouazoubira*, *Mazama nemorivaga*, *Mazama nana*, *Mazama bororo*, *M. americana*, *Blastocerus dichotomus*, *Ozotoceros bezoarticus*, and *O. virginianus* of South American origin. We performed intra- and inter-species comparisons of the obtained satellite DNA sequences and their physical localization on metaphase chromosomes using fluorescence in situ hybridization (FISH). We also searched the obtained sequences for a presence of the 17-bp binding motif for the CENP-B centromeric protein. Finally, we reconstructed phylogenetic trees of the satellite DNA sequences and compared them with the *mt-cyb* gene phylogeny to infer the evolutionary relationships among Neotropical deer species and their position within Cervidae.

## 2. Material and Methods

### 2.1. Species and Samples

Fibroblast tissue cultures prepared according to standard techniques from skin samples of eight Neotropical deer species and available at NUPECCE (Jaboticabal, Brazil) were used in this study for DNA isolation and FISH. No animals were euthanized in this study. The samples are listed in Table 1. To expand our knowledge on satellite DNAs in Cervidae, we also performed an analysis of partial satIII DNA sequence in eight Old world deer species (see Table 1) still missing data on satIII DNA sequence variability. Genomic DNA obtained previously [24] from peripheral lymphocytes was used for the analysis. Taxonomic nomenclature published by Groves and Grubb (2011) was used [5].

### 2.2. Satellite DNA Isolation

Genomic DNA was obtained from fixed suspensions of cultured fibroblasts of the available Neotropical deer using the QIAamp DNA Blood Mini Kit (Qiagen, Hilden, Germany) after washing in PBS. Satellite DNA was isolated from the genomic DNA by PCR amplification using previously published primer sets [24] (Table S1). SatI, satII, and satIV DNA sequences were isolated from all Neotropical deer species. SatIII DNA was obtained only from *M. gouazoubira*, and the satIII DNA internal fragment (satIII-part) was isolated from all remaining Neotropical and Old-World deer samples available for this study. All PCR reactions were performed using Hot Start Combi PPP Master Mix (Top-Bio, Prague, Czech Republic) according to the manufacturer’s instructions. The obtained PCR products were cloned into the pDrive Cloning Vector (Qiagen, Hilden, Germany). Four different clones of each of satI, satII, satIII-part, and satIV DNA were selected in each species on the basis of their Hae III RFLP patterns (recognition site GG*CC) and subjected to sequencing.

### 2.3. Sequence Analysis

All satellite DNA sequences obtained in this study were screened for interspersed repeats using RepeatMasker (http://www.repeatmasker.org). The GC content was calculated using DNA/RNA GC Content Calculator (http://www.endmemo.com). All satellite sequences were also screened for a presence of the 17 bp CENP-B binding motif (NTTCGNNNNANNCGGGN) and the satI for the 31-bp subrepeat unit motif [35,36] using FIMO (version 5.1.0) software (http://meme-suite.org) [37]. The satellite DNA sequences obtained in this study were compared to cervid satellite sequences available in the NCBI database using BLASTN (https://blast.ncbi.nlm.nih.gov) and BLAST2 software was used to assess the sequence homology.

### 2.4. FISH

Cloned satI, satII, satIII, and satIV DNA of *M. gouazoubira* were labelled with Orange- or Green-dUTP (Abbott, Abbott Park, IL, USA) using Nick Translation Reagent Kit (Abbott) to serve as probes for comparative FISH. FISH was performed using standard protocols [19]. Hybridization signals were examined using Zeiss Axio Imager.Z2 fluorescence microscope (Carl Zeiss Microimaging GmbH, Jena, Germany) equipped with appropriate fluorescent filters and the Metafer Slide Scanning System (MetaSystems, Altlussheim, Germany). Images of well-spread metaphase cells were captured and analyzed using ISIS3 software (MetaSystems).

### 2.5. Phylogenetic Analysis

Multiple sequence alignments were constructed in MAFFT 7.474 [38] using the L-INS-i algorithm [39] for each satellite sequence separately. Alignments of cervid satellite DNA contain 1–8% of gaps [24], and gaps can influence phylogenetic reconstruction [40]. To capture phylogenetic information in gaps, the indels in the satI-IV alignments were re-coded to presence/absence data and used as a partition in phylogenetic reconstruction [22]. For the indel partition, gaps in a sequence were coded as 1, and all nucleotides were coded as 0. Optimal substitution models for DNA sequences were selected with the smart model selection algorithm 1.8.4 based on the Bayesian Information Criterion that utilized likelihood estimation implemented in PhyML 3.3 [41,42]. The phylogenetic trees were reconstructed in MrBayes 3.2 [43] in the partitioned analysis, capturing the DNA sequence variation and the phylogenetic information in the indels. The Markov Chains Monte Carlo (MCMC) were run for 2 million generations, sampled every thousandth generation. Two runs of four MCMC were run to ascertain efficient treespace search and check for convergence, following discarding 30% of initial samples as burn-in. The analyses were considered converged when the average standard deviation of split frequencies was <0.01 at the end of the run, potential scale reduction factors were ≈1.000 for each model parameter and frequency of swaps between neighboring chains was between 0.3 and 0.7. The trees were visualized in R [44] with help from packages ape [45], treeio [46], phytools [47], and RColorBrewer [48], where nodes with posterior probability ≥0.95 were considered supported. The trees were rooted at midpoint.

We downloaded cervid reference sequences of the *mt-cyb* gene from the NCBI database to reconstruct a phylogenetic tree from a mitochondrial marker and to compare the satDNA and mtDNA phylogenies. The analysis was performed analogically to the analysis of satDNA sequences, with the difference that the mtDNA marker did not contain gaps and the phylogeny was reconstructed from a single partition containing the DNA sequences.

## 3. Results

### 3.1. Sequence Analysis

In this study, newly obtained satI, satII, satIII-part, and satIV DNA sequences were analyzed in the Neotropical deer species including different *M. americana* cytotypes. The PCR product lengths, GC content, and sequence similarities among the individual satellite DNA clones are displayed in Table 2. Moreover, the satIII-part sequence was isolated and analyzed in *C. elaphus*, *D. dama*, *R. eldii*, *M. reevesi*, *C. capreolus*, *R. tarandus*, and *A. alces*. All satellite DNA sequences obtained in this study were deposited in the NCBI database (accession numbers MW273496–MW273692).

Using RepeatMasker, we did not find any SINE, LINE, or LTR elements in the analyzed satI, satII, and satIV DNA sequences. In all analyzed species, the predicted CENP-B binding motif was detected in the satII DNA starting at position 145–148 bp (Table S2). Eighteen to 21 copies of the 31-subrepeat unit motif were revealed in the satI DNA sequences (Table 2). The sequences of the 31-bp subrepeat unit showed a substantial intra- and interspecies variability. A higher similarity was observed between the subrepeat sequences in a particular satI monomer position in different species than among the subrepeat sequences in the same satI monomer. The 31-bp subrepeat sequence variance and its positions in the satI sequence in the analysed Neotropical deer are shown in Table S3.

Sequence similarity among satellite DNAs of Neotropical deer, Capreolinae, and Cervinae was compared using both sequences obtained in this study, and those available in the NCBI database (Table 3). SatI DNA sequences showed the highest intra- and interspecies variability. A high intra- and interspecies satellite DNA sequence similarity was observed in satII-satIV, even when the Neotropical deer satellite DNA was compared with sequences previously published in Capreolinae and Cervinae (Table 3).

### 3.2. FISH

**Figure 1:**
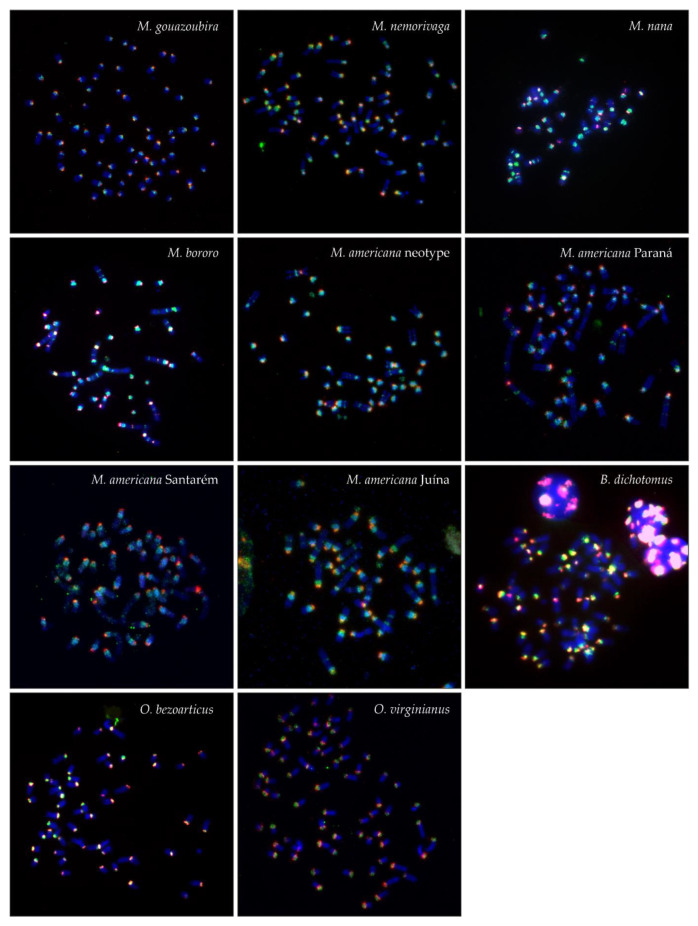
FISH patterns of the satI (**green**) and satII (**red**) DNA probe in the analyzed species.

**Figure 2:**
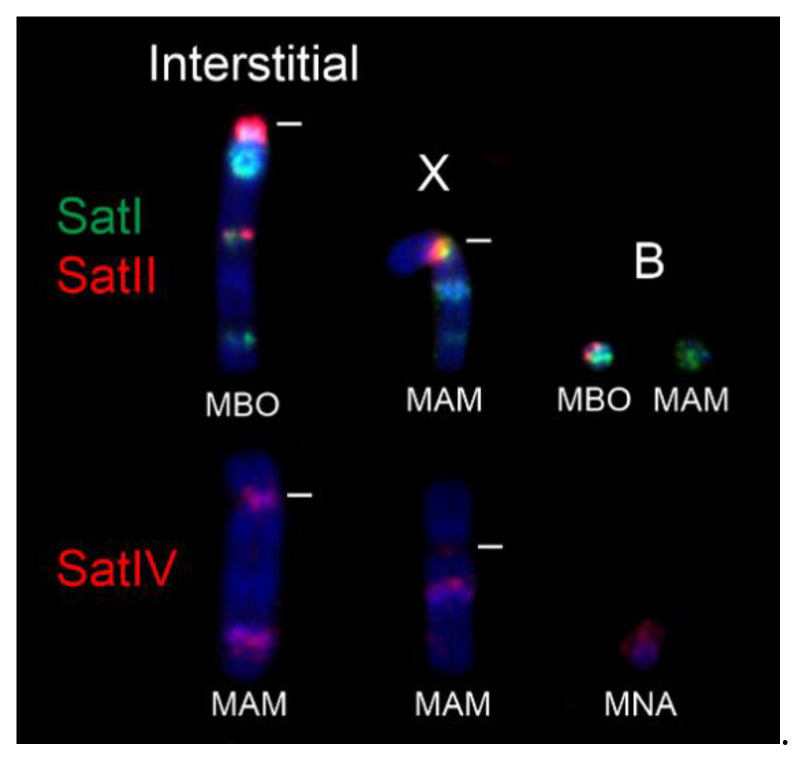
Examples of centromeric and interstitial satI, satII, and satIV signals on autosomes, X chromosomes, and B chromosomes. MBO—*M. bororo*, MNA—*M. nana*, MAM—*M. americana*. Centromeres are indicated by white lines.

**Figure 3:**
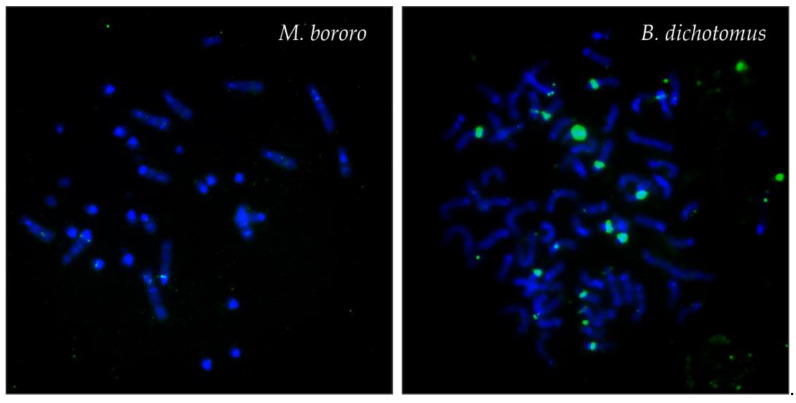
Examples of FISH patterns of the satIII (green) DNA probe in selected species. FISH pattern similar to the results in *M. bororo* was observed in all species except *B. dichotomus*.

**Figure 4:**
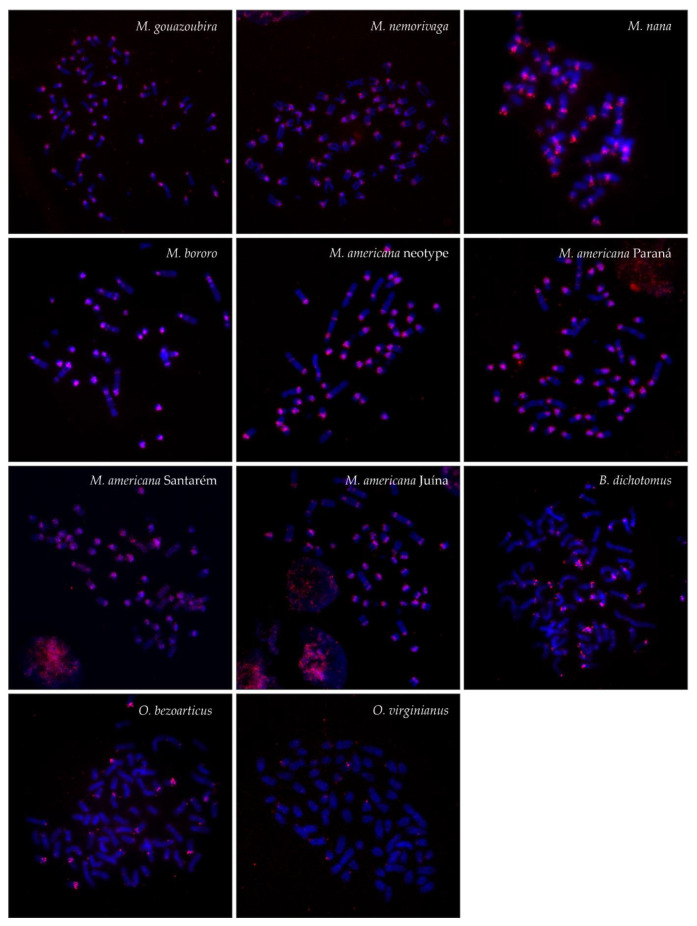
FISH patterns of the satIV (red) DNA probe in the analyzed species.

Probes for satI, satII, satIII, and satIV DNA obtained from *M. gouazoubira* were used for comparative FISH in the 11 Neotropical deer samples. The FISH results are summarized in Table 4 and displayed in Figure 1, Figure 2, Figure 3 and Figure 4.

In general, the MGO satI and satII probe produced centromeric signals on all autosomes (both acrocentric and bi-armed) and on the X chromosome in all analysed Neotropical deer (Figure 1 and Figure 2). In all animals with B chromosomes, the satI probe also marked Bs. Moreover, the satI probe produced interstitial signals on one or more chromosomes in *M. nana*, *M. bororo* and in all four analyzed cytotypes of *M. americana*. Signals of the satII probe were also observed interstitially in *M. nana* and *M. bororo* and on B chromosomes in *M. bororo.* Examples of the interstitial signals and signals on B chromosomes are displayed in detail in Figure 2.

Using the MGO satIII probe, none or only weak subcentromeric signals were detected in the Neotropical Cervidae. The only exception was *B. dichotomus* showing also large signals on several autosomes (Figure 3).

The MGO satIV probe hybridized to large regions of centromeric heterochromatin of all chromosomes in most Neotropical deer species and produced also interstitial signals in *M. bororo* and in all *M. americana* cytotypes. In addition, B chromosomes were marked in *M. nana* (Figure 2 and Figure 4). A different pattern was observed in *B. dichotomus*, *O. bezoarticus*, and the Brazilian *O. virginianus*, only showing very weak centromeric signals of the satIV probe on a few autosomes together with several intense signals in *B. dichotomus* and *O. bezoarticus*.

### 3.3. Phylogenetic Analysis

**Figure 5:**
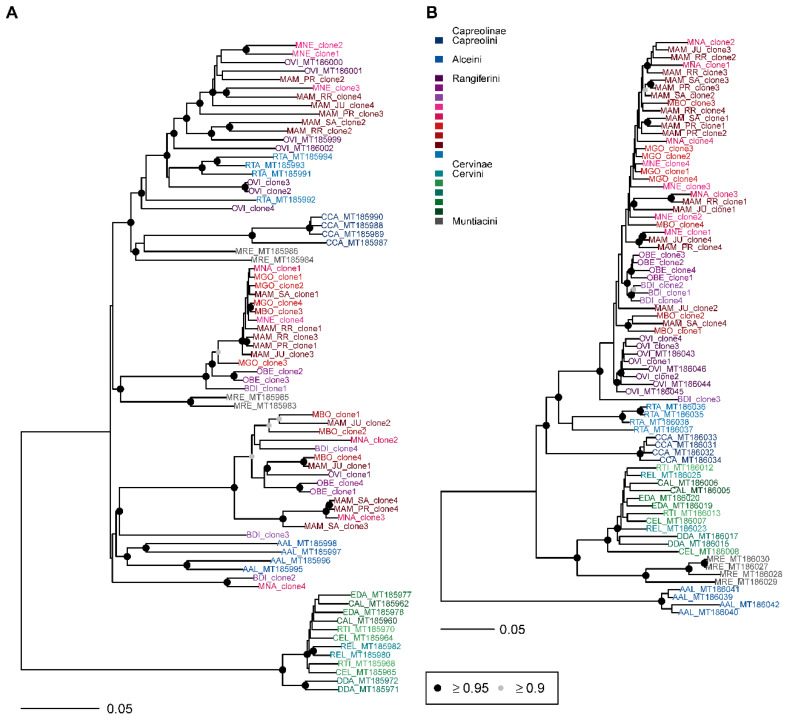
Bayesian phylogenetic trees constructed from cervid satellite sequences. (**A**) SatI, (**B**) satII. AAL—*Alces alces*, BDI —*Blastocerus dichotomus*, CAL—*Cervus albirostris*, CCA—*Capreolus capreolus*, CEL—*Cervus elaphus*, DDA—*Dama dama*, EDA—*Elaphurus davidianus*, MAM—*Mazama americana*, MBO—*Mazama bororo*, MGO—*Mazama gouazoubira*, MNA—*Mazama nana*, MNE—*Mazama nemorivaga*, MRE—*Muntiacus reevesi*, OBE—*Ozotoceros bezoarticus*, OVI—*Odocoileus virginianus*, REL—*Rucervus eldii*, RTA—*Rangifer tarandus*, RTI—*Rusa timorensis*. Circles at nodes signify nodes with posterior probability ≥0.95 (**black**) and ≥0.90 (**grey**). Unmarked nodes were not supported.

**Figure 6:**
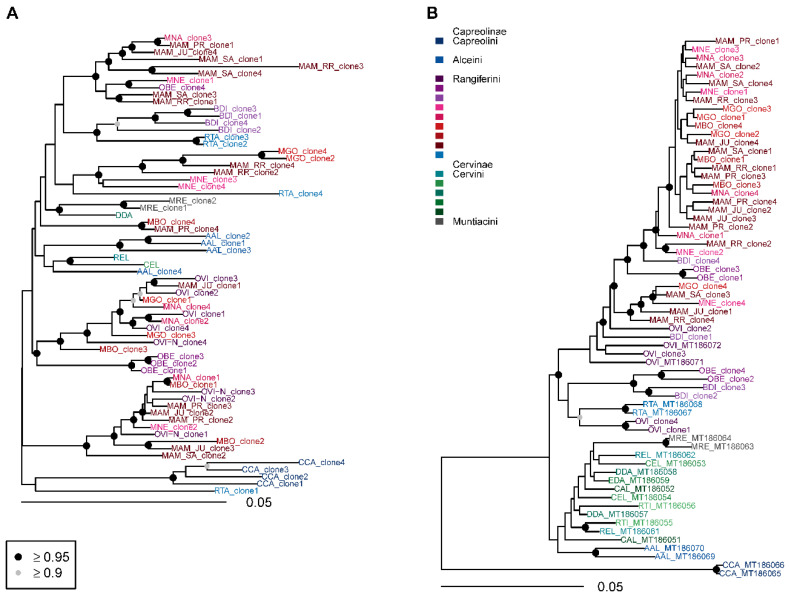
Bayesian phylogenetic trees constructed from cervid satellite sequences. (**A**) SatIII, (**B**) satIV. AAL—*Alces alces*, BDI—*Blastocerus dichotomus*, CAL—*Cervus albirostris*, CCA—*Capreolus capreolus*, CEL—*Cervus elaphus*, DDA—*Dama dama*, EDA—*Elaphurus davidianus*, MAM—*Mazama americana*, MBO—*Mazama bororo*, MGO—*Mazama gouazoubira*, MNA—*Mazama nana*, MNE—*Mazama nemorivaga*, MRE—*Muntiacus reevesi*, OBE—*Ozotoceros bezoarticus*, OVI—*Odocoileus virginianus*, REL—*Rucervus eldii*, RTA—*Rangifer tarandus*, RTI—*Rusa timorensis*. Circles at nodes signify nodes with posterior probability ≥0.95 (**black**) and ≥0.90 (**grey**). Unmarked nodes were not supported.

Together with previously published sequences, multiple sequence alignments consisted of 64 to 76 satI-IV sequences and 18 *mt-cyb* sequences of species from the family Cervidae (Table S4). The smart model selection algorithm suggested the GTR substitution model for the satI and satIII alignments, K80 model for the satII, and HKY model for the satIV and *mt-cyb* alignments. In all satDNA alignments, rate heterogeneity between sites was modelled according to the Γ distribution, and in the *mt-cyb* according to the proportion of invariable sites (Table S4). The satII and satIV phylogenies differentiated currently recognized tribes, with satII sequencing supporting monophyletic groups of Cervini, Muntiacini, Alceini, Capreolini, and Rangiferini (Figure 5B and Figure 6B).

In satI phylogeny, a monophyletic relationship of satellite sequences was supported for Cervini, Alceini, and Capreolini, however Rangiferini and Muntiacini were polyphyletic (Figure 5A). The Neotropical deer satI sequences were diverged and formed three deeply differentiated lineages (Figure 6A). Clones from multiple species (*M. americana*, *M. nemorivaga*, *M. bororo*, *M. nana*, *O. bezoarticus*, *B. dichotomus*) were represented in more than one lineage. In *M. americana*, clones from all sampled regions were present in all three lineages. The first lineage included *Mazama*, *Odocoileus* (both populations), and Holarctic *Rangifer* in incomplete sorting of sequences at the species level, and the lineage formed a sister relationship to *Capreolus* and *Muntiacus*. The second Neotropical deer satI lineage consisted of *Mazama*, *Ozotoceros,* and *Blastocerus* as a sister group to two *Muntiacus* satI sequences. SatI clones from *M. gouazoubira* all belonged to the second lineage. The genera of Neotropical deer grouping in the third satI lineage were also *Mazama*, *Ozotoceros,* and *Blastocerus*, but no clear sister relationship was identified in the satI phylogeny (Figure 6A).

Neotropical deer formed a single supported lineage in the satII sequences with supported monophyly of *Odocoileus*, *Ozotoceros,* and *Blastocerus* (Figure 6B). The latter two taxa formed a paraphyletic sister relationship with respect to *Mazama*. Notably, BDI_clone3 formed a long branch at the base of Neotropical Rangiferini. Similar relationships were retrieved in the satIV phylogeny, but additional clones from all Neotropical genera grouped within the *Mazama* lineage (Figure 6B).

Low divergence in satIII-part (Table 3) resulted in incomplete lineage sorting in Neotropical deer and a polytomy at the deep divergence of Cervidae (Figure 6A).

The *mt-cyb* phylogeny showed monophyletic groups representing the tribes Rangiferini and Cervini (Figure S1). Muntiacini were sister to Cervini, and Capreolini and Alceini diverged rapidly close to the root of the tree. In Neotropical deer, mtDNA phylogeny did not support monophyly of *Mazama* similarly as was shown in the satDNA trees. Instead, *Mazama* were paraphyletic, with *M. nana*, *M. bororo,* and *M. americana* forming a single lineage sister to *O. virginianus*, and *M. gouazoubira* and *M. nemorivaga* forming an unresolved group with *B. dichotomus* and *O. bezoarticus* (Figure S1).

## 4. Discussion

### Sequence Comparisons

Cervidae is a diverse group of species distributed in Eurasia and North and South America. Among them, Neotropical deer species have still been understudied in terms of current taxonomy and phylogenetic relationships [49]. In this study, we performed a comparative sequence and FISH analysis of the four main cervid satellite DNA families (satI-IV) isolated from a variety of Neotropical deer species, including several specific cytotypes.

Our sequence comparisons revealed close relationships among the studied Neotropical deer in satII, satIII and satIV DNA that also showed a high similarity to satellite DNA sequences available in the NCBI database for other species of Capreolinae. Regarding satI DNA, it showed the highest intra- and interspecific variability indicating a fast satI sequence evolution at the time of early divergence of the Capreolinae subfamily. The satI and satII monomer lengths were comparable to previously studied Capreolinae [24]. However, the number of the 31-bp satI internal subrepeat units was lower in the studied Neotropical deer than in other Capreolinae, and closer to that published in Cervini [24]. The general occurrence of this subrepeat throughout many bovid and cervid genomes [21,24,28,50] indicates its possible biological function. This suggestion is also supported by the interspecies similarity in the 31-bp subrepaeat sequences at the individual positions of the satI DNA monomers. This sequence might form a part of a 3D structure involved in protein or siRNA binding during heterochromatin formation, cell division, or transcription regulation. However, further studies are needed to elucidate its functions.

The highest GC content and the presence of the CENP-B binding motif, known to be associated with the centromeric function [51,52], were detected in the satII DNA in all studied Neotropical deer. These findings support the previously published hypothesis that satII DNA might represent the most important satellite DNA family in Cervidae [24] but the satII DNA significance has yet to be confirmed by functional studies. The sequence of the 17-bp CENP-B motif was identical throughout most analyzed Neotropical deer samples indicating a high level of conservation. The most common CENP-B motif sequence detected in this study (TTTGGAGGCAGGCGGGG) contained the published human core recognition sequence (NTTCGNNNNANNCGGGN) [53] with one nucleotide difference (C-G substitution). However, three different one-nucleotide substitutions from the deer core sequence were revealed in *M. bororo*, indicating a surprising CENP-B motif variance in this species that probably originated during its separate evolution.

In Cervidae, satI and satII DNA sequences are highly abundant, occupying 2–35% of the genomic DNA [33]. On the other hand, low copy numbers of satIII and satIV DNA not detectable by FISH were previously reported in many deer species [24]. In this study, we also observed significant differences in the abundance of the satellite DNA families by FISH. The high copy numbers of satI and satII DNA, reflected by large hybridization signals, were in contrast to very weak satIII signals only present on one or a few chromosomes in all studied species except *B. dichotomus*. Regarding satIV DNA, there were significant differences in its abundance between *O. virginianus*, *B. dichotomus* and *O. bezoarticus* on one side, and species of the genus *Mazama* on the other, the latter showing large satIV DNA blocks. The observed interspecies differences in the satellite DNA abundance at the relatively high sequence similarity of the repeat units can be related to the satellite DNA evolution. It is generally accepted that satellite DNAs are formed by a fast amplification of monomers existing in ancestral genomes [54]. As suggested by a library model, related species share a common collection of satellite DNAs, which vary in their abundance, with specific sequences being differentially amplified in individual species [55,56,57]. Satellite DNA arrays are probably formed by a mechanism of rolling circle replication with subsequent further amplification by unequal crossing over [58,59,60,61]. The existing sequences are then diversified by mutations, which spread through the genome by mechanisms of concerted evolution, leading to the formation of species- and chromosome-specific satellite sequences [13,62,63,64]. Accordingly, interspecies differences in satellite DNA abundance and sequences were found to be highly consistent with species phylogeny [16,22,24,56,63,65].

In this study, the satII DNA sequence phylogeny well corresponds to the mtDNA (*mt-cyb* gene) phylogeny (Figure S1) and the published taxonomic divergences in Cervidae [24]. The satII phylogeny differentiated the currently recognized tribes, suggesting monophyletic origin of Cervini, Muntiacini, Alceini, Capreolini, and Rangiferini. In Neotropical deer, *O. bezoarticus* was closely related to *B. dichotomus*, and both Neotropical and Nearctic populations of *O. virginianus* formed one lineage without defined intraspecific relationships. SatII and satIV sequences of *Mazama* showed unresolved relationships both at the level of species differentiation as well as at the genus level. Incomplete lineage sorting of *Mazama* species with respect to other Neotropical deer consistently occurs in satellite DNA (this study), mtDNA phylogenies (Figure S1 and [66,67]), and even with the molecular and morphological data combined [68]. Neotropical deer, as descendants of Nearctic ancestors that arrived to South America during the Great America Biotic Interchange between late Miocene and late Pleistocene [69], diverged in an explosive radiation, forming morphologically well-defined but genetically unresolved genera. As in other Neotropical mammals, morphological convergence in genetically diverged taxa could be attributed to ecological adaptations to specific niche partitioning in newly colonized regions [70,71]). In this study, the explosive radiation in Neotropical deer can be best observed in the satI sequence phylogeny. At least four distinct lineages first began to diverge at the time of the split of Capreolinae. Genera *Rangifer*, *Capreolus*, and *Alces* each retained one lineage of satI sequences, but in Neotropical deer, satI sequences diversified three times independently.

The presence of genomic satellite DNA arrays can facilitate the formation of chromosome rearrangements and thus karyotype and species evolution [29,72,73]. The deer chromosome evolution from the ancestral karyotype (2n = 70) was driven by centric and tandem fusions at the simultaneous reduction in the chromosome number [9]. Moreover, the existence of centric fusion polymorphisms previously described in the genus *Mazama* indicate that chromosome fusions represent an important source of the recent and ongoing karyotype evolution of this taxon [74,75,76]. Despite the predominantly peri/centromeric location of the satellite DNA, we also observed interstitial satI, satII, and satIV FISH signals in *Mazama* species with highly reduced chromosome numbers (*M. nana*, *M. bororo*, *M. americana* cytotypes). Similar finding of interstitial satellite DNA signals at the tandem fusion sites, and even their co-localization with telomeric sequences was previously reported in muntjacs [30,32,77,78,79].

In *M. americana*, a multiple sexual system resulting from evolutionary X-autosomal fusions was previously described [12,80]. Among deer, the XY1Y2 system was found also in the genus *Muntiacus* [81,82]. It is known that sex-autosomal translocations are usually associated with a disturbed process of X chromosome inactivation and with a meiotic disruption [83,84,85]. However, heterochromatin blocks intercalated between the gonosomal and autosomal parts of the rearranged sex-autosomes can serve as effective barriers for spreading of somatic X-chromosome inactivation in females and for regulation of meiotic processes in males [86,87,88]. In our study, the presence of intercalated heterochromatin in the X-autosomal fusion region suggested by a distinct DAPI band was proved by detection of satI, less frequently satII and satIV hybridisation signals in all *M. americana* cytotypes. As discussed in the previous paragraph, also these interstitial satellite DNA signals probably map to a historical fusion site and represent former centromeric heterochromatin of the autosome fused to the ancestral X chromosome.

Another interesting karyotype feature of *Mazama* species analyzed in this study is the presence of B chromosomes [7,12,75,80]. B chromosomes are supernumerary, mitotically unstable chromosomes that were described in some animal, plant, and fungal species [89]. Their numbers vary in different individuals and even individual cells of the organism, and, despite a presence of duplicated coding genes detected in Bs in some species [90,91], their biological function is unknown. A recent study based on comparative FISH and next-generation sequencing of B chromosomes in two deer species, *Capreolus pygargus* (Capreolini) and *M. gouazoubira* (Rangiferini) demonstrated an independent origin of B chromosomes in these species, and their different evolutionary history [92]. In all animals carrying B chromosomes in our study, the Bs showed FISH signals of the satI DNA probe. The independent divergent lineages observed in Neotropical deer in the satI phylogeny might be attributed to satI sequences on the B chromosomes. Moreover, the satII DNA signals were detected on Bs in *M. bororo*. This might indicate either an independent origin or a more primitive stage of the B chromosomes in *M. bororo*, which have not yet led to an evolutionary satII DNA sequence degeneration in this species.

## 5. Conclusions

The Neotropical deer species show high intra- and interspecies satellite DNA sequence similarities indicating close evolutionary relationships. The high satellite DNA abundance probably stands behind the cervid karyotype differentiation driven by centric and tandem fusions at the simultaneous reduction of the chromosome number.

## Figures and Tables

**Table 1:** Species analyzed in this study.

Species	Latin Name	Abbr. ^a^	2n
Brown brocket deer	*Mazama gouazoubira*	MGO	2n = 70 + B
Amazonian brown brocket deer	*Mazama nemorivaga*	MNE	2n = 69 + B
Brazilian dwarf brocket deer	*Mazama nana*	MNA	2n = 39 + Bs
Small red brocket deer	*Mazama bororo*	MBO	2n = 33 + Bs
Red brocket deer-cytotype Paraná	*Mazama americana*	MAM-PR	2n = 53 + Bs
Red brocket deer-cytotype Santarém	*M. americana*	MAM-SA	2n = 51 + Bs
Red brocket deer-cytotype Juína	*M. americana*	MAM-JU	2n = 45 + Bs
Red brocket deer-neotype from Roraima ^b^	*M. americana*	MAM-RR	2n = 46 + Bs
Marsh deer	*Blastocerus dichotomus*	BDI	2n = 66
Pampas deer	*Ozotoceros bezoarticus*	OBE	2n = 68
White-tailed deer of Brazilian origin	*Odocoileus virginianus*	OVI	2n = 70
Red deer	*Cervus elaphus*	CEL	2n = 68
Fallow deer	*Dama dama*	DDA	2n = 68
Eld’s deer	*Rucervus eldii*	REL	2n = 58
Chinese muntjac	*Muntiacus reevesi*	MRE	2n = 46
Roe deer	*Capreolus capreolus*	CCA	2n = 70
Reindeer	*Rangifer tarandus*	RTA	2n = 70
Moose	*Alces alces*	AAL	2n = 68
White-tailed deer of North American origin	*Odocoileus virginianus*	OVI-N	2n = 70

^a^ Abbreviation; ^b^ An animal from the region Roraima cytogenetically similar to the previously described neotype [10] but showing a heterozygous centric fission.

**Table 2:** Characteristics of the satI-IV sequences based on four clones of each satellite DNA analyzed in each sample of Neotropical deer (see text for abbreviations of species names).

Species	SatI				SatII			SatIII			SatIV		
	Length (bp)	GC Content (%)	Similarity (%)	No. of 31–bp Units	Length (bp)	GC Content (%)	Similarity (%)	Length (bp)	GC Content (%)	Similarity (%)	Length (bp)	GC Content (%)	Similarity (%)
MGO	910	51	96–99	19	579–581	67	97–99	583	53–56	89–98	727	45	97–99
MNE	910–913	51–53	76–92	19	578–580	65–67	92–96	580–583	55–58	90–93	726–727	44–45	96–99
MNA	904–919	46–51	75–84	20	575–579	64–67	90–93	579–583	56–59	89–97	727–728	45–46	98–99
MBO	910–917	49–51	76–92	18	578–579	64–66	92–94	579–583	55–58	90–94	727	45	99–100
MAM_PR	904–913	47–54	75–85	19	579	65–67	94–97	579–583	56–59	90–98	727	45	98–99
MAM_SA	904–910	47–51	72–94	18	578–579	64–67	92–98	581–583	56–59	90–95	727	44–46	97–99
MAM_JU	909–917	49–53	74–94	18	579–581	66–67	92–95	578–583	56–60	90–96	726–727	45–46	96–99
MAM_RR	908–910	51–52	77–98	20	575–579	64–67	93–96	580–583	54–59	86–93	727	45	97–99
BDI	911–919	48–50	77–80	21	579–580	63–67	88–98	579–584	56	92–97	726–737	45	93–97
OBE	911–917	48–51	76–98	18	580	67–68	96–99	582–583	55–58	91–99	727	45–46	92–99
OVI	907–915	48–51	74–99	19	578–579	66–67	97–98	581–584	55–56	94–97	727	44–46	95–99

**Table 3:** Satellite DNA sequence similarity among Neotropical deer, Capreolinae, and Cervinae (Cervini and Muntjacini).

Species	SatI			SatII			SatIII–Partial			SatIV		
	Neotropical Deer	Capreolinae	Cervinae	Neotropical Deer	Capreolinae	Cervinae	Neotropical Deer	Capreolinae	Cervinae	Neotropical Deer	Capreolinae	Cervinae
MGO	75–99	76–81	79–83	88–99	77–97	77–84	85–99	83–95	87–93	93–99	93–96	85–97
MNE	71–99	73–89	76–83	86–99	77–97	75–84	86–99	85–98	89–93	92–99	92–96	85–97
MNA	71–99	69–80	73–83	85–97	73–95	75–82	87–99	85–98	89–94	93–99	93–96	85–97
MBO	73–99	71–80	73–83	87–97	76–95	75–82	86–99	85–98	89–94	93–99	93–96	85–97
MAM_PR	72–99	73–89	74–83	87–99	77–96	76–84	87–99	85–98	89–94	93–99	93–96	85–96
MAM_SA	72–99	72–86	74–83	87–99	76–97	74–84	87–97	84–95	88–93	93–99	93–96	85–97
MAM_JU	72–99	70–86	73–83	86–97	76–96	74–83	88–99	85–98	89–94	93–99	93–96	85–97
MAM_RR	72–99	74–88	76–83	86–97	74–96	75–83	85–97	83–92	86–93	93–99	93–97	85–97
BDI	73–95	72–80	75–83	85–98	75–97	72–84	85–93	85–92	89–94	91–98	92–96	84–96
OBE	73–96	72–81	75–83	88–98	78–97	76–84	86–97	85–92	89–94	92–97	92–96	84–95
OVI	72–92	72–87	73–84	79–98	79–98	77–85	86–98	85–95	89–93	93–98	93–97	85–97

**Table 4:** Fluorescence in situ hybridization (FISH) patterns of the MGO satI-IV probes in Neotropical deer.

Species	2n	FN	B	SatI	SatII	SatIII	SatIV
MGO	70	70	+	all autosomes, X, Bs	all autosomes, X	a single autosome, weak	all autosomes, X
MNE	69	72	+	all autosomes, X, Bs	all autosomes, X	a few autosomes, weak	all autosomes
MNA	39	58	+	all autosomes, X, Bs, interstitial	all autosomes, X, interstitial	a few autosomes, weak	all autosomes, X, Bs, interstitial
MBO	33	46	+	all autosomes, X, Bs, interstitial	all autosomes, X, Bs, interstitial	a few autosomes, weak	most autosomes, X, interstitial
MAM PR	53	56	+	all autosomes, X, Bs, interstitial	all autosomes, X	a few autosomes, weak	all autosomes, X, interstitial
MAM SA	51	56	+	all autosomes, X, Bs, interstitial	all autosomes, X,	a few autosomes, weak	all autosomes, interstitial
MAM JU	45	48	+	all autosomes, X, Bs, interstitial	all autosomes, X	a few autosomes, weak	all autosomes, X, interstitial
MAM RR	46	51	+	all autosomes, X, Bs, interstitial	all autosomes, X	a few autosomes, weak	all autosomes, interstitial
BDI	66	74	-	all autosomes, X	all autosomes, X	a few autosomes, big or weak	a few autosomes, big or weak
OBE	68	74	-	all autosomes, X	all autosomes, X	a few autosomes, weak	a few autosomes
OVI	70	74	-	all autosomes, X	all autosomes, X	a few autosomes, weak	a few autosomes

## Data Availability

The data presented in this study are available in the article and supplementary material.

## Supplementary Materials

The following are available online at https://www.mdpi.com/2073-4425/12/1/123/s1: Table S1. Primers and annealing temperatures for satellite DNA isolation. Table S2. Positions and sequences of the CENP-B binding motif in individual satII DNA clones. Table S3. The 31-bp subrepeat sequence variance and its positions in the satI DNA sequence. Table S4. Multiple sequence alignment composition and selected substitution models of cervid satellite and mitochondrial sequences. Figure S1. Bayesian phylogenetic tree constructed from cervid *mt-cyb* sequences. AAL—*Alces alces*, BDI—*Blastocerus dichotomus*, CAL—*Cervus albirostris*, CCA—*Capreolus capreolus*, CEL—*Cervus elaphus*, DDA—*Dama dama*, EDA—*Elaphurus davidianus*, MAM—*Mazama americana*, MBO—*Mazama bororo*, MGO—*Mazama gouazoubira*, MNA—*Mazama nana*, MNE—*Mazama nemorivaga*, MRE—*Muntiacus reevesi*, OBE—*Ozotoceros bezoarticus*, OVI—*Odocoileus virginianus*, REL—*Rucervus eldii*, RTA—*Rangifer tarandus*, RTI—*Rusa timorensis*. Circles at nodes signify nodes with posterior probability ≥0.95 (black) and ≥0.90 (grey). Unmarked nodes were not supported.
