# Supplementary material for: Satellite DNA in Neotropical Deer Species
Source: Genes (Basel). 2021 Jan 19;12(1):123. doi: 10.3390/genes12010123 (PMC7835801; doi:10.3390/genes12010123)
Supplement: Supplementary file 1 [file genes-12-00123-s001.zip › Table_S1.pdf]

Table S1. Primers and annealing temperatures for satellite DNA isolation.

Target	Primers		Annealing temperature
SatI	CAAGACGAAAGGATGTCTGAATCC	GGTGTCCATTCCACTTGAGGC	58oC
SatII	CTGTGAGTGTGGAGCCCCGAGC	GTGGGAAGAGGCAGAGCCGACC	64oC
SatIII	GAGGTGTGACCGTGACAGGACCC	CGAGGCTGGGATGTTGTGAGAAGC	64oC
SatIII-partial	ACAGATGGAGAACATCCCTCTGG	GTGAATACGAAAAGGACTGTGGG	57oC
SatIV	TTGATATTAGGTGATTGGATGGG	AAGATGTCAGAACTTCAGGTTTGC	57oC
