# Supplementary material for: Satellite DNA in Neotropical Deer Species
Source: Genes (Basel). 2021 Jan 19;12(1):123. doi: 10.3390/genes12010123 (PMC7835801; doi:10.3390/genes12010123)
Supplement: Supplementary file 1 [file genes-12-00123-s001.zip › Table_S2.pdf]

Table S2. Positions and sequences of the CENP-B binding motif in individual satII DNA clones.

Sequence Name	Start	p-value	q-value	Matched Sequence
MGO_clone1	146	1.34e-05	0.00988	TTTGGAGGCAGGCGGGG
MGO_clone2	146	1.34e-05	0.00988	TTTGGAGGCAGGCGGGG
MGO_clone3	148	1.34e-05	0.00988	TTTGGAGGCAGGCGGGG
MGO_clone4	146	1.34e-05	0.00988	TTTGGAGGCAGGCGGGG
MNE_clone2	146	1.34e-05	0.00988	TTTGGAGGCAGGCGGGG
MNE_clone3	146	1.34e-05	0.00988	TTTGGAGGCAGGCGGGG
MNE_clone4	146	1.34e-05	0.00988	TTTGGAGGCAGGCGGGG
MNA_clone1	146	1.36e-05	0.00563	TTTGGAGGCAGGCGGGG
MNA_clone2	146	1.36e-05	0.00563	TTTGGAGGCAGGCGGGG
MNA_clone3	146	1.36e-05	0.00563	TTTGGAGGCAGGCGGGG
MNA_clone4	146	2.95e-05	0.00978	TTTGGAGACAGGCGGGG
MBO_clone1	146	0.000262	0.0362	TTTGCAGGCAGGCGGGG
MBO_clone2	146	0.000262	0.0362	TTTGCAGGCAGGCGGGG
MBO_clone3	146	1.36e-05	0.00563	TTTGGAGGCAGGCGGGG
MBO_clone4	146	0.000153	0.0307	TTCGGAGGCAGGCGGGG
MAM_PR_clone1	146	1.34e-05	0.00988	TTTGGAGGCAGGCGGGG
MAM_PR_clone2	146	1.34e-05	0.00988	TTTGGAGGCAGGCGGGG
MAM_PR_clone4	146	1.34e-05	0.00988	TTTGGAGGCAGGCGGGG
MAM_SA_clone1	146	1.34e-05	0.00988	TTTGGAGGCAGGCGGGG
MAM_SA_clone2	146	1.34e-05	0.00988	TTTGGAGGCAGGCGGGG
MAM_JU_clone2	146	1.34e-05	0.00988	TTTGGAGGCAGGCGGGG
MAM_JU_clone3	146	1.34e-05	0.00988	TTTGGAGGCAGGCGGGG
MAM_JU_clone4	146	1.34e-05	0.00988	TTTGGAGGCAGGCGGGG
MAM_RR_clone1	146	1.34e-05	0.00988	TTTGGAGGCAGGCGGGG
MAM_RR_clone2	146	2.87e-05	0.0204	TTTGGATGCAGGCGGGG
MAM_RR_clone3	146	1.34e-05	0.00988	TTTGGAGGCAGGCGGGG
MAM_RR_clone4	146	1.34e-05	0.00988	TTTGGAGGCAGGCGGGG
BDI_clone1	146	1.36e-05	0.00563	TTTGGAGGCAGGCGGGG
BDI_clone2	146	1.36e-05	0.00563	TTTGGAGGCAGGCGGGG
BDI_clone3	146	1.36e-05	0.00563	TTTGGAGGCAGGCGGGG
BDI_clone4	146	1.36e-05	0.00563	TTTGGAGGCAGGCGGGG
OBE_clone2	146	1.34e-05	0.00988	TTTGGAGGCAGGCGGGG
OBE_clone3	146	1.34e-05	0.00988	TTTGGAGGCAGGCGGGG
OBE_clone4	146	1.34e-05	0.00988	TTTGGAGGCAGGCGGGG
OVI_clone1	146	1.34e-05	0.00988	TTTGGAGGCAGGCGGGG
OVI_clone2	145	1.34e-05	0.00988	TTTGGAGGCAGGCGGGG
OVI_clone4	146	1.34e-05	0.00988	TTTGGAGGCAGGCGGGG
