# Supplementary material for: Satellite DNA in Neotropical Deer Species
Source: Genes (Basel). 2021 Jan 19;12(1):123. doi: 10.3390/genes12010123 (PMC7835801; doi:10.3390/genes12010123)
Supplement: Supplementary file 1 [file genes-12-00123-s001.zip › Table_S4.pdf]

Table S4. Multiple sequence alignment composition and selected substitution models of cervid satellite sequences.

Satellite sequence	Number of sequences	Alignment length (bp)	Proportion of gaps (%)	Substitution model	α
satI	76	934	2.8	GTR + Γ	2.6
satII	76	690	7.4	K80 + Γ	3.5
satIII	65	598	1.4	GTR + Γ	0.7
satIV	64	747	1.3	HKY + Γ	1.6
mt-cyb gene	18	1140	0	HKY + I	I = 0.61

Γ - rate heterogeneity between sites modelled with the Γ distribution, α - shape parameter of the Γ distribution.
