# Supplementary figures and images for: Satellite DNA in Neotropical Deer Species
Source: Genes (Basel). 2021 Jan 19;12(1):123. doi: 10.3390/genes12010123 (PMC7835801; doi:10.3390/genes12010123)

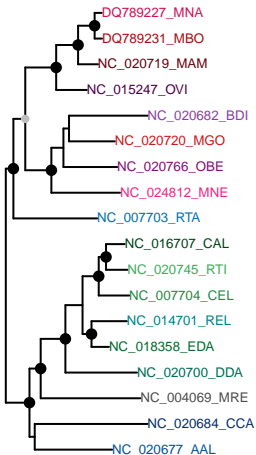

- Capreolinae
  - Capreolini
  - Alceini
  - Rangiferini
- Cervinae
  - Cervini
- Muntiacini

● ≥ 0.95    ● ≥ 0.9

— 0.05

Supplement: Supplementary file 1 [file genes-12-00123-s001.zip › Figure S1.pdf]
